# Impaired TNF*α*-induced A20 expression in E1A/Ras-transformed cells

**DOI:** 10.1038/sj.bjc.6605352

**Published:** 2009-10-13

**Authors:** H-L Huang, W-C Yeh, M-Z Lai, C Mirtsos, H Chau, C-H Chou, S Benchimol

**Affiliations:** 1 The Campbell Family Institute of Breast Cancer Research, Ontario Cancer Institute, University Health Network and Department of Medical Biophysics, University of Toronto, 620 University Avenue, Toronto, Ontario, Canada; 2 Institute of Molecular Biology, Academia Sinica, Taiwan, ROC; 3 Department of Bioscience Technology, College of Health Science, Chang Jung Christian University, Tainan, Taiwan, ROC; 4 Department of Biology, York University, Toronto, Ontario, Canada

**Keywords:** TNF*α*, A20, Bcl-3, NF-*κ*B, Ras, E1A

## Abstract

**Background:**

Tumour necrosis factor (TNF) is capable of activating the cell death pathway, and has been implicated in killing transformed cells. However, TNF also activates survival signals, including NF-*κ*B activation and the subsequent expression of anti-apoptotic genes, leading to protection against TNF toxicity.

**Methods:**

In this study, we show that, although untransformed mouse embryonic fibroblasts (MEFs) were resistant to TNF killing, E1A/Ras-transformed MEFs were susceptible to extensive apoptosis induced by TNF. The key factors for determining TNF sensitivity were explored by comparing wild-type and E1A/Ras-transformed MEFs.

**Results:**

TNF signalling to NF-*κ*B and to its target genes such as I*κ*B*α* seemed to be mostly intact in E1A/Ras-transformed cells. Instead, the induction of A20 was completely abolished in E1A/Ras-transformed MEFs, although A20 is known to be NF-*κ*B dependent. Reintroduction of A20 into E1A/Ras-transformed MEFs rescued these cells from TNF-induced death and reduced the formation of the FADD/caspase-8 complex. This impaired A20 induction in E1A/Ras MEFs was not because of the stabilisation of p53 or a defective TNF-induced p38 and Jun N-terminal kinase (JNK) signalling. Consistently, we found a reduced A20 promoter activity but normal NF-*κ*B activity in TNF-treated E1A/Ras MEFs. However, Bcl-3 seemed to have a role in the transactivation of the A20 promoter in E1A/Ras cells.

**Conclusions:**

Our results suggest that specific inhibition of certain survival factors, such as A20, may determine the sensitivity to TNF-induced apoptosis in transformed cells such as E1A/Ras MEFs.

Tumour necrosis factor (TNF) is a pro-inflammatory cytokine that induces cell death in sarcoma cells but not in untransformed mouse embryonic fibroblasts (MEFs) (Carswell *et al*, 1975). Studies of downstream signalling pathways, particularly those mediated by TNF receptor 1 (TNFR1), reveal that TNF can induce both death and survival pathways (Hsu *et al*, 1996; Mak and Yeh, 2002). Exactly how these death and survival signals interact to determine cell fate remains ambiguous.

Activation of NF-*κ*B by TNF and expression of specific proteins downstream of NF-*κ*B seem to have a crucial role in TNF-induced survival signals (Rubin *et al*, 1988; Beg and Baltimore, 1996; Van Antwerp *et al*, 1996; Wang *et al*, 1996). Several NF-*κ*B-inducible, antiapoptotic proteins have been identified, including TNFR-associated factors (TRAFs), inhibitors of apoptosis (IAPs), Bcl-2 family proteins, platelet-derived growth factor B (PDGF-B), and A20, which seem to act in a cooperative manner for cell survival (Opipari *et al*, 1992; Wang *et al*, 1998; Yeh *et al*, 1999; Baetu *et al*, 2001). The IAPs and Bcl-2 family proteins are direct antagonists of the activation of caspases and apoptotic machinery, and the terminal cytotoxic processes induced by TNF are thereby shut down. Other proteins, such as PDGF-B, do not seem to directly interfere with the death machinery, and their exact anti-apoptotic mechanisms remain to be elucidated.

A clue to understanding how cell death or survival outcomes are determined after TNF stimulation comes from studies of the TNF-induced protein complex assembly. It was established that binding of TNF initiates protein–protein interactions between TNFR1 and the TNFR-associated death domain protein (TRADD). TRADD in turn recruits receptor-interacting protein (RIP) and TRAF2 for NF-*κ*B and survival signals, or recruits the Fas-associated death domain protein (FADD) for apoptotic execution (Hsu *et al*, 1995, 1996; Chen and Goeddel, 2002; Mak and Yeh, 2002). This laid the foundation for TNF signalling dichotomy, supported by genetic evidence that deficiency of one set of signals results in the dominance of the other pathway (Yeh *et al*, 1997, 1998).

Recently, a more detailed analysis of protein signalling complexes induced by TNF/TNFR1 binding reveals that two major complexes can potentially be assembled after TNF stimulation (Micheau and Tschopp, 2003). Complex I, composed of TNFR1, TRADD, RIP1, TRAF2, and other accessory proteins, is rapidly assembled in the lipid rafts of plasma membrane upon TNF binding, and is responsible for activating NF-*κ*B. Within 1 hour of binding, the cytoplasmic components of Complex I then dissociate from TNFR1, allowing the interaction of TRADD/RIP1/TRAF2 with FADD and caspase-8, with a subsequent formation of Complex II. Notably, Complex II is readily formed on TNF stimulation in Ras-transformed human fibrosarcoma cells (Legler *et al*, 2003; Micheau & Tschopp, 2003). In these cells, the activation of caspase-8 within Complex II is the major turning point for cell fate in response to TNF.

In this study, we analysed the signalling in normal TNF-resistant MEFs and TNF-sensitive MEFs that were transformed by E1A and Ras. Frequently found in human cancers, constitutively active Ras induces senescence, but cooperates with other oncoproteins such as adenoviral E1A and c-Myc to transform primary human and rodent cells (Ruley, 1983; Serrano *et al*, 1997). Comparison between untransformed and transformed MEFs revealed a specific defect of A20 induction by TNF stimulation in E1A/Ras-transformed cells, despite the normal NF-*κ*B activation and I*κ*B*α* induction. In addition, ectopic expression of A20 in transformed MEFs rescued the cells from TNF-induced death, suggesting that A20 may regulate the original signalling complex assembled by TNF stimulation (Wertz *et al*, 2004). Furthermore, we identified a role of Bcl-3 in TNF-induced A20 expression, which was dysregulated in E1A/Ras-transformed MEFs. Our results uncovered a novel and potentially critical imbalance within transformed cells because of the lack of key survival factor(s) such as A20 and/or possibly A20-like proteins, thereby allowing the formation of a death-inducing complex when stimulated with TNF.

## Materials and methods

### Cell culture and E1A/Ras-transformed MEFs

**Figure 1:**
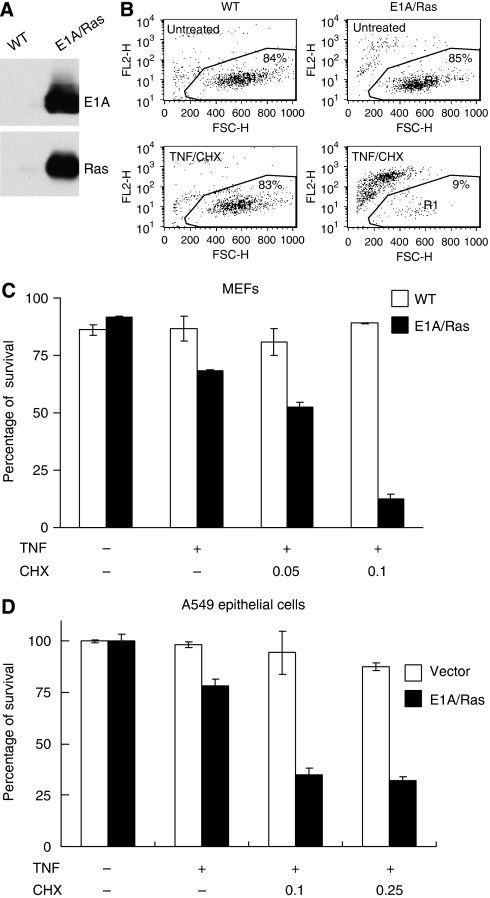
Selective TNF sensitivity in E1A/Ras-transformed MEFs. (**A**) Total cell lysates from WT or E1A/Ras-transformed MEFs were immunoblotted with E1A and Ras antibodies. (**B** and **C**) E1A/Ras-transformed MEFs were sensitive to TNF-induced cell death. WT or E1A/Ras-transformed MEFs were treated with 10 ng ml^−1^ TNF plus 0, 0.025, or 0.1 *μ*g ml^−1^ CHX (TNF plus 0.1 *μ*g ml^−1^ CHX for Figure 1B) for 16 h, and the percentage of viable cells was analysed by flow cytometry. (**D**) A549 epithelial cells were first transduced with vector- or E1A/Ras-containing viruses; thereafter, cells were left untreated or treated with 10 ng ml^−1^ TNF plus 0, 0.1, or 0.25 *μ*g ml^−1^ CHX (as indicated) for 24 h. Viable cells were detected by MTS assay, and the relative percentage of viable cells (percentage of survival) was calculated as follows: [mean OD (treated)/mean OD (untreated)] × 100.

MEFs were cultured in Dulbecco's modified Eagle's medium supplemented with 10% fetal bovine serum (Gibco, Grand Island, NY, USA) and 50 *μ*M *β*-mercaptoethanol (Gibco). To prepare transformed MEFs, E1A and Ras were expressed using pLPC or pBabe retroviral vectors, which were kindly provided by Drs Scott W Lowe (Cold Spring Harbor, NY, USA) and Miria Soengas (University of Michigan Comprehensive Cancer Center) as described (Berube *et al*, 2005). Briefly, MEFs were infected with retrovirus-containing culture supernatant from transfected Phoenix packaging cells. For pLPC (McCurrach *et al*, 1997), cells grown in puromycin (2 *μ*g ml^−1^) medium for 7 days were selected. The p53^+/+^ and p53^−/−^E1A/Ras MEFs were also gifts from Dr Scott Lowe (Lowe *et al*, 1994). For the A20 or Bcl-3 stable expression MEFs, pBabe retroviral vector, and pBabeA20 or pBabeBcl-3 were used for retroviral production; then E1A/Ras MEFs (for A20) or wild-type MEFs (for Bcl-3) were infected with retrovirus and selected using puromycin as described above. For A549 E1A/Ras cells (Figure 1D), human A549 epithelial cells were cultured in 10% serum-containing F-12 medium (Gibco) and were infected with E1A/Ras gene-containing retrovirus culture supernatant. TNF sensitivity was tested 2–6 days after transduction.

### Plasmids

Human Bcl-3 cDNA (Open BioSystems, Huntsville, AL, USA) was subcloned into pBabe retroviral vector digested with *Bam*HI and *Sal*I. For the antisense Bcl-3 used in Figure 8B, an inverted orientation of the Bcl-3 fragment (757–232), as previously described (Brasier *et al*, 2001), was generated by RT–PCR and cloned into pcDNA4. Both murine p50 and p52 cDNA were generated by RT–PCR using total cDNA as template from wild-type MEFs. The PCR primers used are as follows: p50 5′-gcagacgatgatccctacgg-3′ and 5′-ctatatggtgccatgggtgacc-3′; p52 5′-gacaattgctacgatccaggc-3′ and 5′- tcacgtctagaaccggccatc-3′. After sequence confirmation, both cDNA fragments were inserted into pBabe retroviral vector (Figure 8A) or p4SV40 vector (Figure 8C; CMV promoter in pcDNA4 was replaced with the SV40 promoter from pBabe).

### Cell death assay

After incubation with 10 ng ml^−1^ recombinant TNF*α* (R&D system, Minneapolis, MN, USA) plus 0 to 0.1 *μ*g ml^−1^ CHX (Sigma) for 9–16 h, attached and suspended MEFs cells were collected and resuspended in phosphate-buffered saline (PBS) containing 1 *μ*g ml^−1^ propidium iodide (Sigma, St Louis, MO, USA). The percentage of viable cells was detected and analysed by flow cytometry (FACSCalibur, Becton Dickenson, Mountain View, CA, USA). For the detection of apoptosis by fragmented nuclei observation, MEFs were cultured on coverglasses coated with poly-L-lysine (Sigma). After TNF*α* and CHX treatment for 14 h, cells were washed, fixed with 3.7% formaldehyde/PBS, stained with 4′, 6-diamidino-2-phenyindole (DAPI; Sigma), and finally observed using a confocal laser-scanning microscope (Zeiss). For A549 E1A/Ras cells in Figure 1D, the relative percentage of viable cells was detected and analysed by MTS assay (Promega, Madison, WI, USA), according to the manufacturer's instructions.

### Northern blot analysis

Total RNA from MEFs was extracted using TRIzol reagent (Invitrogen, Carlsbad, CA, USA), resolved on 1% denaturing formaldehyde agarose gels, and transferred to Hybond N membranes (GE Healthcare, Milwaukee, WI, USA) (Yeh *et al*, 2000). cDNA probes for A20, I*κ*B*α*, and GAPDH were used to hybridise with the blots. For Figure 6B, wild-type MEFs were pretreated for 1 h with 25 *μ*M of the p38 inhibitor SB203580 or the JNK inhibitor II (Calbiochem, La Jolla, CA, USA), before being treated with TNF*α*.

### Immunoprecipitation (i.p.) and western blot analysis

After TNF treatment, MEFs were dissolved in RIPA buffer (PBS containing 0.1% SDS, 1% NP-40, and 0.5% Sodium deoxycholate) with protease inhibitors (Roche Biochemicals, Indianapolis, IN, USA) for FADD i.p. reactions. After removal of debris by centrifugation, lysates were immunoprecipitated with FADD antiserum (kindly provided by D Goeddel, Z Cao, and G Chen, Tularik, South San Francisco), and were washed 5 times. Immunoprecipitates were resuspended in sample buffer, subjected to 10% SDS–PAGE, and immunoblotted with FADD (Upstate, Lake Placid, NY, USA) and Caspase-8 (Alexis, San Diego, CA, USA) antibodies for the same blots. The following antibodies were also used for straight western blot analysis: cFLIP (Apotech, San Diego, CA, USA), I*κ*B (Cell Signaling, Beverly, MA, USA or Santa Cruz, Santa Cruz, CA, USA), Bcl-2, c-IAP2 (Santa Cruz), XIAP (BD Biosciences, Mountain View, CA, USA), phospho-p38, phospho-JNK, phosphor-ERK, ERK (Cell Signaling), p38, and JNK2 (Santa Cruz).

### Gel mobility shift assays and oligonucleotide pull-down assays

Gel mobility shift assays were performed as previously described (Yeh *et al*, 1997). Nuclear extracts were purified from untreated or TNF*α*-and CHX-treated MEFs , and consensus NF-*κ*B probes were used for these assays. Similar to gel mobility shift assays, the NF-*κ*B probe-associated NF-*κ*B family members from nuclear extracts were checked by oligonucleotide pull-down assays (Bundy and McKeithan, 1997; Budunova *et al*, 1999; Corn *et al*, 2005) as follows: We used biotinylated oligonucleotides of the NF-*κ*B binding site from human A20 promoter. The sequences were (sense) 5′-GTGACTTTGGAAAGTCCCGTGGAAATCCCCGGGC-3′ and (antisense) 5′-GCCCGGGGATTTCCACGGGACTTTCCAAAGTCAC-3′. Equal amounts of both nucleotides were first annealed as dsDNA probe. Nuclear extracts prepared as above were mixed with biotinylated dsDNA probe on ice for 1 h, then incubated for another 2 h with streptavidin-agarose beads (Sigma) in binding buffer (20 mM HEPES pH7.8, 50 mM KCl, 0.2 mM EDTA, 1.5 mM MgCl2, 1 mM DTT, 0.25% Triton, and 10% glycerol). After this pull-down reaction, beads were washed with binding buffer and *κ*B probe-bound proteins were subjected to 9% SDS–PAGE and immunoblotted with the following antibodies: p65, p50, Bcl-3 (Santa Cruz), p100/52 (Cell Signaling), and Hsc70 for the same blots.

### Luciferase activity assay

HEK293 cells or MEFs were seeded into 24-well (for 293 cells) or 12-well (for MEFs) plates 1 day before transfection. The same amounts of A20-Luc reporter plasmid (kindly provided by Dr Rivka Dikstein (Ainbinder *et al*, 2002)) and pCMV*β*Gal were co-transfected with 2- to 4-fold of plasmids (such as Bcl-3, as indicated in Figures 6, 7 and 8) into HEK293 cells using Superfect (Qiagen, Indianapolis, IN, USA) or into MEFs using Fugene 6 (Roche, Indianapolis, IN, USA). Two days later, cells were left untreated or treated 6 h with human or murine TNF*α* 10 ng ml^−1^ (for 293 or MEFs, respectively) in the absence of CHX. After the cells were washed with PBS, luciferase activity in cell lysates was detected using the Luciferase Assay System (Promega) according to the manufacturer's instructions, and was normalised to *β*-galactosidase activity (Yeh *et al*, 1997, 2000).

## Results

### Expression of many anti-apoptotic proteins is unaffected in TNF-sensitive MEFs transformed by E1A/Ras

Interaction of FADD and caspase-8 with TNFR signalling proteins is an essential feature in transformed fibrosarcoma cells on TNF stimulation (Micheau and Tschopp, 2003). For comparison between TNF-sensitive transformed MEFs and TNF-resistant wild-type MEF cells, we generated transformed MEFs by overexpression of E1A and Ras (Figure 1A). E1A/Ras-transformed cells were more sensitive to cell death induced by TNF, and this cytotoxicity became even more prominent if cells were treated with TNF plus a trace amount of cycloheximide (CHX, 0.025 or 0.1 *μ*g ml^−1^), as shown in Figures 1B and 1C. Untransformed MEFs were not affected by the treatment of TNF plus 0.1 *μ*g ml^−1^ CHX (Figure 1B), whereas the viability of E1A/Ras MEFs was severely impaired.

To further extend our findings, we tested whether this phenomenon is also true in epithelial cells (Figure 1D), particularly those with established Ras oncogenic activity. We chose to use the A549 cell line, which was reported to be insensitive to TNF-induced toxicity in an earlier study (Sugarman *et al*, 1985). After retrovirus-mediated vector or E1A/Ras expression, A549 epithelial cells were treated with TNF alone or with TNF plus a trace amount of CHX. Consistent with the MEF system, A549 E1A/Ras cells were more sensitive to TNF killing when compared with cells expressing vector alone (Figure 1D).

**Figure 2:**
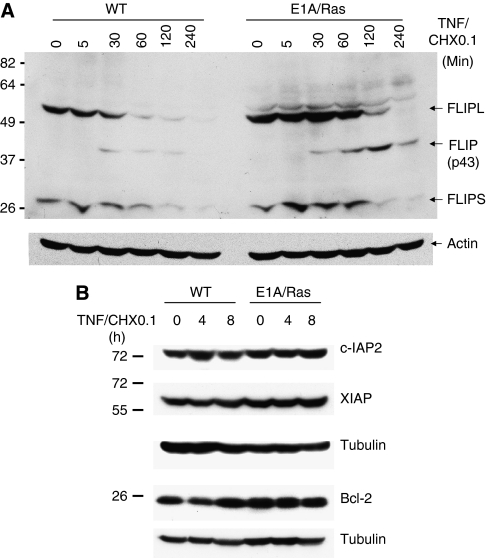
Expression of some key anti-apoptotic proteins in TNF-stimulated E1A/Ras-transformed cells. E1A/Ras-transformed cells were left untreated or treated with 10 ng ml^−1^ TNF plus 0.1 *μ*g ml^−1^ CHX for 5–240 min (**A**) or for 4 h and 8 h (**B**) as indicated. Cell lysates were subjected to 10% SDS–PAGE, then immunoblotted with cFLIP (**A**) or with c-IAP2, Bcl-2, and XIAP antibodies (**B**). The same blots were also immunoblotted with actin or tubulin as loading controls.

To investigate what could potentially account for TNF sensitivity in E1A/Ras-transformed *vs* control wild-type MEFs, we examined the expression of various anti-apoptotic proteins. cFLIP is a protein that directly antagonises TNF- and other death factor-induced apoptosis (Yeh *et al*, 2000) by interfering with the activation of caspase-8. We found that expressions of cFLIP_L_, cFLIP_S_, and the processed derivative of cFLIP (p43) did not seem to be defective in E1A/Ras-transformed cells (Figure 2A). Instead, by an unknown mechanism, the expression of cFLIP_L_ and cFLIP (p43) was slightly higher in transformed MEFs than in wild-type MEFs. We further examined the expression of other anti-apoptotic proteins including c-IAP2, XIAP, and Bcl-2. The expressions of c-IAP2, XIAP, and Bcl-2, in the presence or absence of TNF stimulation, were similar between untransformed and E1A/Ras-transformed MEFs (Figure 2B), suggesting that the enhanced TNF susceptibility of E1A/Ras MEFs was not because of the downregulation of these anti-apoptotic factors.

### TNF-induced A20 expression, but not NF-*κ*B, is impaired in E1A/Ras MEFs

**Figure 3:**
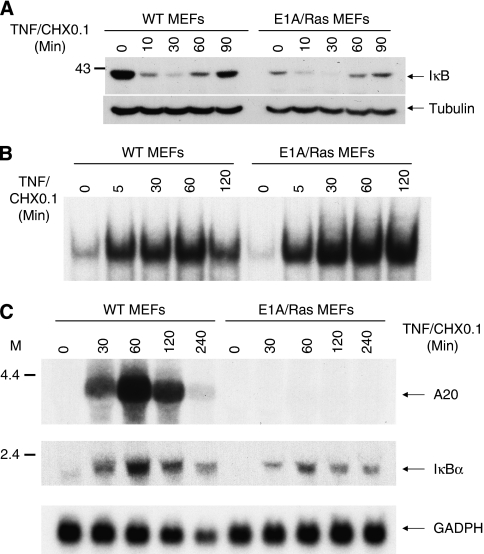
Deficiency of A20 induction in E1A/Ras-transformed MEFs. (**A** and **B**) NF-*κ*B activation in TNF-treated WT or E1A/Ras-transformed cells. (**A**) Total lysates from WT or E1A/Ras MEFs were immunoblotted for detection of I*κ*B degradation. (**B**) Gel mobility shift assays were performed. Nuclear extracts were collected from TNF-treated MEFs, incubated with ^32^P-labelled NF-*κ*B consensus-binding oligonucleotides, and then resolved in acrylamide gel. (**C**) Repression of TNF-induced A20 expression in E1A/Ras-transformed cells. WT or E1A/Ras-transformed MEFs were untreated or treated with 10 ng ml^−1^ TNF plus 0.1 *μ*g ml^−1^ CHX for 30–240 min. Total RNA was purified and 20 *μ*g of RNA was separated on 1% agarose gel, followed by northern blot analysis using isotope-labelled A20 cDNA as a probe. The same blot was stripped and hybridised with I*κ*B*α* and GAPDH probes.

As the expression of survival genes induced by TNF is mostly NF-*κ*B dependent, we examined whether the increased TNF sensitivity in E1A/Ras MEFs was caused by a perturbation of NF-*κ*B activation. As shown in Figure 3A, TNF-induced I*κ*B degradation proceeded normally in E1A/Ras MEFs, as compared with that in wild-type cells. Measurement of DNA binding by gel mobility shift assays also revealed that TNF-induced formation of the NF-*κ*B–DNA complex was not defective in E1A/Ras-transformed cells (Figure 3B). A slight increase in NF-*κ*B DNA-binding activity was instead observed in E1A/Ras MEFs stimulated with TNF.

We next examined the expression of two NF-*κ*B target genes induced by TNF. Although TNF-induced I*κ*B*α* mRNA expression was unaffected in E1A/Ras-transformed cells, A20 mRNA induction was totally abolished in these transformed cells (Figure 3C). The defect was not restricted to a specific E1A/Ras-transformed cell line, as similar results were found in several E1A/Ras-transformed MEF lines that we generated (data not shown), or in the lines obtained from other laboratories (for example, Dr Scott Lowe) (see 6B). The defect was also evident in E1A/Ras-transformed MEFs treated with TNF alone (independent of CHX; data not shown, also see 6B and 7B). As A20 is implicated in the protection against TNF-induced apoptosis, the specific defect in A20 induction may contribute to the TNF sensitivity observed in E1A/Ras MEFs.

### Reconstitution of A20 in E1A/Ras-transformed MEFs protects cells from TNF toxicity

**Figure 4:**
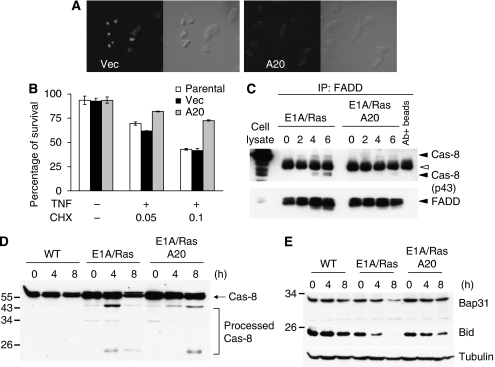
A20 rescues E1A/Ras-transformed MEFs from TNF-induced cell death. Empty vector or A20 was transduced into E1A/Ras MEFs by the retroviral expression system. After selection with puromycin, cells were left untreated or treated with 10 ng ml^−1^ TNF plus 0.1 *μ*g ml^−1^ CHX for 12 h (**A**) or plus 0.025 or 0.1 *μ*g ml^−1^ CHX for 9 h (**B**). (**A**) Both vector and A20 stable E1A/Ra MEFs were stained with DAPI, and the chromatin condensation inside the nuclei was checked by confocal microscopy. (**B**) Suspended and attached E1A/Ras MEFs were collected and stained with PI for death assay as described in Materials and Methods. (**C**–**E**) Cells as indicated in figures were left untreated or treated with TNF plus 0.1 *μ*g ml^−1^ CHX for 4 or 8 h. Cell lysates were immunoprecipitated with FADD antiserum. Immunoprecipitates or total cell lysates (as labelled) were subjected to 10% SDS–PAGE, and then analysed by western blotting using caspase-8 and FADD antibodies (**C**). Expression of Caspase-8, Bap31, and Bid in total cell lysates was detected by immunoblotting (**D**–**E**).

The process of E1A/Ras transformation is complicated and it is likely that multiple events and changes are involved. To investigate whether the absence of A20 induction has a key role in sensitising cells to TNF-induced apoptosis, we restored the A20 expression in E1A/Ras MEFs using retrovirus transfection. Compared with parental or empty-vector-expressing cells, A20 stable expression significantly rescued E1A/Ras-transformed MEFs from TNF-induced cell death (Figure 4A and 4B). The same result was observed in three independent A20-expressing E1A/Ras MEF cell lines and in their controls (data not shown). We next examined whether the formation of a complex containing FADD and caspase-8 differed between these MEF lines. Assembled FADD-associated protein complexes were examined by immunoprecipitation, followed by western blotting. In addition to the full-length caspase-8, the processed caspase-8 p43/41 was also associated with FADD (Figure 4C), as reported previously (Micheau and Tschopp, 2003), in E1A/Ras-transformed MEFs. However, the TNF-induced death signalling complex that co-immunoprecipitated with FADD was decreased in A20-expressing E1A/Ras MEF cells (Figure 4C), suggesting that A20 has a key role in guarding E1A/Ras-transformed MEFs against TNF-induced cell death.

Furthermore, we observed that caspase-8 was processed in E1A/Ras MEFs after TNF treatment, in contrast to wild-type MEF (Figure 4D). Reconstitution with A20 delayed the degradation of caspase-8 in E1A/Ras MEFs. Caspase-8 activation led to the cleavage of BAP31 (Ng *et al*, 1997) and Bid (Mak and Yeh, 2002), which was also suppressed by the reconstitution of A20 (Figure 4E). Taken together, our results suggest that the deficiency of A20 induction in TNF-stimulated E1A/Ras MEFs is a key factor contributing to their susceptibility to cell death mediated by TNF. As A20 reconstitution did not completely revert cells to normal, it is possible that other unidentified survival factors are also involved in E1A/Ras MEFs.

### JNK, p38, or p53 is not required to suppress A20 expression in E1A/Ras-transformed MEFs

**Figure 5:**
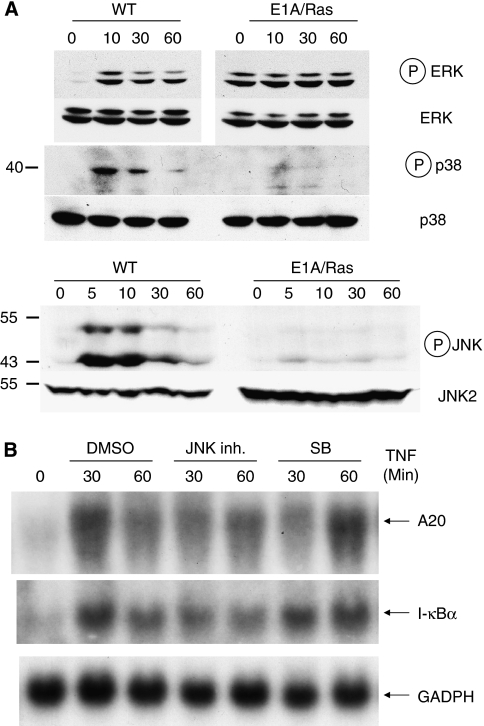
Abrogation of TNF-induced A20 expression is independent of the reduced p38 or JNK signalling in E1A/Ras-transformed MEFs. (**A**) Defective p38 and JNK signalling in E1A/Ras-transformed MEFs. Cells as indicated in figures were left untreated or treated with TNF plus 0.1 *μ*g ml^−1^ CHX for 5–60 min. Expression of phosphorylated or total ERK, p38, and JNK in both cell lysates was detected by immunoblotting using antibodies as indicated. (**B**) WT MEFs were pretreated with 25 *μ*M of the p38 kinase inhibitor SB203580 or JNK inhibitor; thereafter, cells were untreated or treated with 10 ng ml^−1^ TNF plus 0.1 *μ*g ml^−1^ CHX. Expression of A20 or I-*κ*B mRNA was detected by northern blot analysis, as shown in Figure 3C.

We next investigated MAPK signalling pathways downstream of TNF in these transformed MEFs. Intriguingly, TNF-induced p38 and JNK phosphorylations were also reduced in E1A/Ras-transformed cells (Figure 5A), reminiscent of the phenomenon observed in RIP−/− MEFs (Devin *et al*, 2000). In contrast, phosphorylations of ERK and Akt induced by TNF seemed similar between wild-type and E1A/Ras MEFs (Figure 5A, data not shown), except that basal phospho-ERK was enhanced in untreated E1A/Ras MEFs probably because of constitutive Ras activity.

To further examine whether defective p38 or JNK signals affect A20 inducibility, we stimulated wild-type MEFs with TNF in the absence or presence of JNK or p38-specific inhibitors. As shown in Figure 5B, inhibition of either the p38 or JNK pathway did not completely shut down but delayed A20 induction by TNF. In addition, co-treatment of both inhibitors only partially inhibited A20 expression (data not shown). These data suggested that although p38 and JNK pathways may have a role in A20 expression, the deficiency of A20 induction in TNF-stimulated E1A/Ras MEFs was not entirely dependent on the defects of p38 and JNK in these cells.

**Figure 6:**
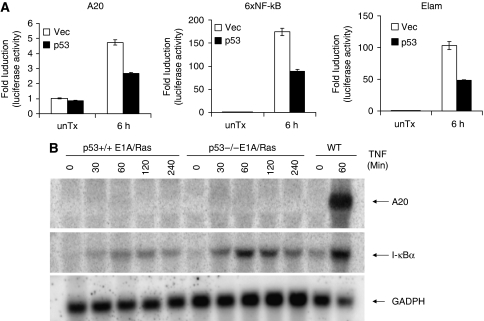
Inhibition of TNF-induced A20 expression is not regulated by p53. (**A**) A20, 6 × NF-*κ*B, or ELAM luciferase reporter plasmids were co-transfected with vector or with p53 into HEK293 cells for 24 h. All cells were co-transfected with pCMV*β*Gal plasmid to monitor transfection efficiency. Twenty-four hours after transfection, cells were left untreated or treated with 10 ng ml^−1^ human TNF*α* for 6 h. Cell lysates were then collected and used for reporter assay. The results were normalised with *β*-galactosidase activity. (**B**) p53^+/+^ or p53^−/−^ E1A/Ras MEFs were treated with 10 ng ml^−1^ TNF alone, as indicated, and used for northern blot analysis as described in Figure 3C.

Another possibility could be that expression of A20 was suppressed by factors induced or upregulated by E1A/Ras transformation. As E1A/Ras triggers signals that stabilise the p53 protein (Lowe *et al*, 1993, 1994), we thus investigated whether p53 inhibits the transcriptional activation of the *A20* promoter. As shown in Figure 6A, TNF-induced A20 promoter activity was suppressed in the presence of p53. However, p53 overexpression in this reporter/transfection setting also suppressed the activation of the E-selectin promoter (ELAM), with NF-*κ*B as the major transcriptional element, and an artificial promoter containing six tandem NF-*κ*B-binding sites (6 × NF-*κ*B) (Yeh *et al*, 2000; Komarova *et al*, 2005). Therefore, p53 suppresses the activation of the A20 promoter, as well as that of other *κ*B-containing promoters. If A20 expression was primarily inhibited by p53, most NF-*κ*B-mediated transactivation should also be blocked in E1A/Ras MEFs.

To further delineate the role of p53 in the inhibition of A20 expression, we examined the induction of A20 by TNF stimulation in both p53^+/+^ and p53^−/−^ E1A/Ras-transformed MEFs. The NF-*κ*B target gene, I*κ*B*α*, was induced by TNF in E1A/Ras MEF cells with or without p53 (Figure 6B). A small increase in I*κ*B*α* expression was detected in p53-deficient E1A/Ras MEFs, suggesting a relief of p53-mediated inhibition of I*κ*B*α* expression. However, expression of A20 was not restored in transformed cells that lacked p53 (Figure 6B). These results suggested that p53 is not the major factor responsible for the suppressed A20 induction in E1A/Ras-transformed MEFs.

### The role of Bcl-3 in the regulation of A20 expression

**Figure 7:**
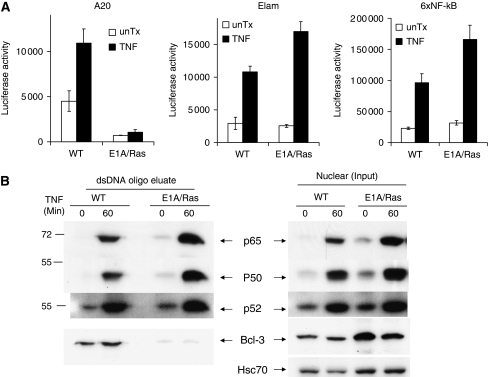
Defective A20 promoter activity in E1A/Ras-transformed MEFs. (**A**) A20, 6 × NF-*κ*B, or ELAM luciferase reporter plasmids (250 ng) were co-transfected with pCMV*β*Gal plasmid (250 ng) into WT or E1A/Ras-transformed MEFs for 40 h. Thereafter, cells were treated with 10 ng ml^−1^ mTNF alone for another 8 h. Reporter assay was performed as described. The relative luciferase activity was normalised with *β*-galactosidase activity. (**B**) Bcl-3 cannot associate with an NF-*κ*B site in the A20 promoter in E1A/Ras-transformed MEFs. Similar to EMSA, after MEFs were treated with mTNF alone for 1 h, nuclear extracts as indicated were incubated with biotinylated dsDNA oligonucleotide. After being purified by SA beads, both the *κ*B site binding proteins and 1/20 of the crude nuclear extracts (as input control) were subjected to 9% SDS–PAGE for immunoblotting. The same blots were immunoblotted with anti-p65, p50, p52, or Bcl3 antibodies, as well as with Hsc70 antiserum.

As the transcriptional activation of the *A20* gene primarily depends on NF-*κ*B (Ainbinder *et al*, 2002), the defective A20 induction in E1A/Ras MEFs with a normal NF-*κ*B activation was unexpected (Figure 3). As shown in Figure 7A and consistent with Figure 3, activation of NF-*κ*B-driven promoters (ELAM and 6 × NF-*κ*B) was in fact slightly higher in E1A/Ras MEFs than in wild-type controls after TNF treatment. On the other hand, both untreated (basal level) and TNF-induced activation of the A20 promoter were severely compromised in E1A/Ras MEFs (Figure 7A). The control of certain target genes may involve other NF-*κ*B family members, in addition to p65 and p50 (Natoli *et al*, 2005; Hoffmann *et al*, 2006; Hayden and Ghosh, 2008). We next investigated, by comparing wild-type and E1A/Ras cells, which the NF-*κ*B member may interact with the *κ*B binding site derived from A20 promoter using oligonucleotide pull-down assay. Interestingly, we found that A20 promoter binding by Bcl-3, but not by p65, p50, p52 (Figure 7B), or c-Rel (data not shown), was specifically defective in E1A/Ras MEFs. The amount of Bcl-3 in nuclei of both wild-type and E1A/Ras cells was similar. Intriguingly, Bcl-3 showed a predominantly nuclear localisation in the presence or absence of TNF in MEFs (data not shown) – similar to some earlier reports (Bours *et al*, 1993; Zhang *et al*, 2000) but different from its expression in keratinocytes (Massoumi *et al*, 2006).

**Figure 8:**
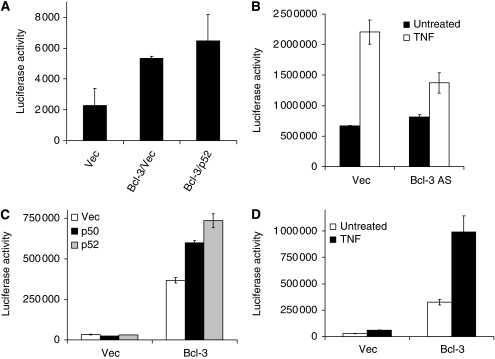
Transactivation of the A20 promoter by Bcl3. The reporter assay for this figure was performed as described in Figures 6 and 7. (**A**) As indicated in the figure, wild-type MEFs were transfected with pBabe vector, pBabeBcl-3, or pBabep52 (200 ng each) expression plasmids, plus A20 luciferase reporter (100 ng)/pCMV*β*Gal plasmids (100 ng) for 48 h. Thereafter, reporter assay was performed. (**B**) A total of 293 cells were transfected with vector or Bcl3 antisense (640 ng) plus A20 luciferase reporter (80 ng)/pCMV*β*Gal plasmids (80 ng) for 40 h, then the cells were left untreated or treated with hTNF 10 ng ml^−1^ (as indicated in the figure) for another 8 h. Luciferase assay was then performed. (**C** and **D**) Bcl3 stable MEFs were generated by the retroviral transduction system using wild-type MEFs as described in Materials and Methods. After selection with puromycin, MEFs were transfected with p4SV40 vector, p4SV40-p50 or p52 (400 ng) expression plasmids (as indicated), plus A20 luciferase reporter (100 ng)/pCMV*β*Gal plasmids (100 ng) for 48 h before the luciferase assay was performed (**C**). (**D**) Similar to Figure 8C, after transfection with A20 luciferase reporter (250 ng)/pCMV*β*Gal plasmids (250 ng) for 40 h, Bcl3 stable MEFs were left untreated or treated with 10 ng ml^−1^ mTNF for another 8 h. Then luciferase assay was performed and relative luciferase activity was normalised with *β*-galactosidase activity.

Bcl-3 was found to be a co-activator of p50 and p52 for the transactivation of cyclin D1, Bcl-2, GATA-3, etc. (Westerheide *et al*, 2001; Viatour *et al*, 2003; Corn *et al*, 2005; Massoumi *et al*, 2006). To further examine whether Bcl-3 is involved in the regulation of A20 expression, a transient overexpression of Bcl-3 was performed and activation of A20 promoter activity was observed in transfected cells measured by luciferase activity (Figure 8A). In contrast, antisense Bcl-3 seemed to reduce TNF-induced A20 promoter activity in 293 cells (Figure 8B). We next examined a Bcl-3 stably expressing MEF line and found that A20 promoter activity was increased with or without TNF stimulation (Figure 8C and D). Similar to other Bcl-3 target genes, co-transfection of p52 or p50 further enhanced A20 promoter activity as shown in Figures 8A and 8C. Taken together, Bcl-3 is capable of regulating the expression of A20 and may be one of the key factors involved in differential TNF responses from wild-type and E1A/Ras-transformed cells.

## Discussion

**Figure 9:**
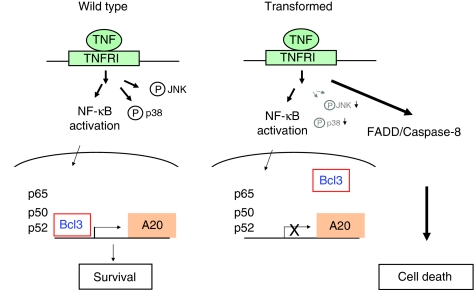
A hypothetical model of this study. See text for details.

E1A/Ras-transformed cells are known to undergo cell death induced by various stimuli, including chemotherapeutics and TNF (Figure 1) (Ames *et al*, 1990; Yeh *et al*, 1998; Locksley *et al*, 2001). In this study, we demonstrated this phenomenon in MEFs and epithelial cells (Figure 1D). Exactly why these cells are more susceptible to TNF remains an important open question. In this study, we compared untransformed and E1A/Ras-transformed MEFs, and found that although TNF-induced NF-*κ*B activation was intact, induction of A20 was impaired in E1A/Ras-transformed cells (Figure 9). This defect of A20 likely has a key role in the susceptibility of these transformed cells to TNF, as reconstitution of A20 partly protected E1A/Ras MEFs from TNF-induced cell death. Interestingly, oestrogen receptor signalling can inhibit A20 expression, which correlates with the sensitivity of breast cancer cells to tamoxifen with a better prognosis (Vendrell *et al*, 2007). In addition, tamoxifen is capable of augmenting cytotoxicity to TNF or to its related death factor TRAIL in breast cancer cells (Tiwari *et al*, 1991; Lagadec *et al*, 2008). Another interesting study (not cancer related) demonstrated the antiapoptotic role for A20 in pancreatic *β* cells, as A20 is possibly the most highly regulated anti-apoptotic gene stimulated by cytokines (Liuwantara *et al*, 2006). This is especially important for the correlation between diabetes and cell death in insulin-producing *β* cells.

The exact mechanism of E1A/Ras suppression of A20 induction remains to be determined. No significant defect in the activation of NF-*κ*B, a major transcription factor dictating A20 expression, was observed in E1A/Ras-transformed MEFs (Figure 3A and 3B). There was instead a small increase in NF-*κ*B–DNA complex formation in E1A/RasMEFs (Figure 3B). p53, which is important for Ras-induced senescence and chemotherapy-induced apoptosis, is thought to be stabilised in E1A/Ras MEFs (Lowe *et al*, 1993, 1994). In our experiments, p53 inhibited NF-*κ*B activation (Figure 4A), as previously reported (Komarova *et al*, 2005). However, as p53 deficiency did not restore A20 induction in E1A/Ras MEFs (Figure 4B), p53 does not seem to be the major factor that inhibits A20 expression, although a lesser role for p53 in suppressing A20 expression cannot be completely excluded. Along the same line, overexpression of PML, a target and a collaborator of p53, inhibits TNF-induced A20 expression in osteosarcoma cells, and this inhibition correlates with the sensitivity of these cells to TNF-induced apoptosis (Wu *et al*, 2002).

In addition to p53, we examined for other defective signalling pathways that may affect A20 induction in E1A/Ras-transformed MEFs. Interestingly, compared with wild-type MEFs, TNF-induced p38 and JNK phosphorylation seemed to be reduced in E1A/Ras-transformed MEFs (Figures 5A and 9). When wild-type cells were pretreated with inhibitors against JNK or p38, A20 induction was mildly reduced or delayed. It is possible that both JNK and p38, probably in combination with other factors/pathways, have a role in the impaired induction of A20 in E1A/Ras-transformed MEFs. However, A20 expression does not seem to strictly depend on these MAPK signalling pathways.

A20 has been implicated as a survival factor that antagonises TNF-induced death (Opipari *et al*, 1992; Jaattela *et al*, 1996; De Valck *et al*, 1999; Lademann *et al*, 2001). A20-knockout mice are runted and highly sensitive to LPS- or TNF-induced toxicity *in vivo* (Lee *et al*, 2000; Boone *et al*, 2004). Cells lacking A20 are hypersensitive to TNF-induced cell death. It is possible that induction of A20 by TNF represents a feedback inhibition event, and A20 may interfere with further death signal progression by interacting with protein(s) involved in TNF signalling. Indeed, A20 has been shown to interact with TRAF2 and NEMO in the TNF-signalling complex (Zhang *et al*, 2000). A20 also contains dual enzymatic activities of de-ubiquitination (from its OTU domain) and E3 ubiquitin ligase (zinc finger) (Wertz *et al*, 2004; Heyninck and Beyaert, 2005). It has been proposed that A20 is able to remove K63-linked ubiquitin from RIP, which deactivates RIP and prevents it from associating with the signalling protein complex, and is also able to ubiquitinate RIP at the K48 residue, which targets it for proteasome-mediated degradation.

The action of A20 is further complicated by its role in regulating NF-*κ*B activity induced by TNF and other pro-inflammatory stimuli. A20-deficient animals and cells show phenotypes of elevated NF-*κ*B activities triggered by TNF or Toll-like receptors (Lee *et al*, 2000; Boone *et al*, 2004), and overexpression of A20 inhibits NF-*κ*B activation induced by TNF (Heyninck and Beyaert, 2005). Some of the key factors for NF-*κ*B activation, including RIP and TRAF6, may be substrates for A20, and A20 may control NF-*κ*B signalling activities by targeting these key proteins for ubiquitination. Aberrant NF-*κ*B activation has been associated with the development of tumours (Karin, 2006). For example, loss of CYLD, a key de-ubiquitinating enzyme that controls NEMO function, has been linked to a predisposition to cylindroma (Brummelkamp *et al*, 2003; Trompouki *et al*, 2003). It is thus possible that loss of A20 may result in deregulated NF-*κ*B activity, which contributes to the abnormality of transformed cells. E1A/Ras MEF cells exhibited moderately enhanced and prolonged NF-*κ*B activation when challenged with TNF (Figure 3B). How the aberrant NF-*κ*B activity affects these transformed MEF cells remains to be investigated.

Hence if A20-deficient cells respond to TNF with a normal or higher activity of NF-*κ*B survival signal, why are they more sensitive to TNF-induced apoptosis? This is an intriguing question that remains to be investigated. One key factor determining the life and death of cells after TNF stimulation is the successful assembly of a death signalling complex (Complex II as referred to by a report published by J Tschopp's group) (Micheau and Tschopp, 2003). The formation of TNF-induced pro-apoptotic complex II depends on the components of the earlier signalling complex (Complex I). RIP is a component of TNF-induced signal complex I, but it also participates in death signalling complex II formation. Targeting RIP is expected to affect both NF-*κ*B signal through complex I and apoptosis induction through complex II. Because A20 regulates RIP activity, there exists a possibility that the phenotype seen with E1A/Ras MEFs in this study is because of a de-regulation of RIP. The failure of TNF to induce A20 in E1A/Ras-transformed cells may therefore lead to abnormal NF-*κ*B activation and enhanced assembly of the death signalling complex. Studies are ongoing to delineate the molecular link between A20 and complex II formation.

Another interesting question is how different NF-*κ*B family members cooperate to regulate a diversity of NF-*κ*B target genes, including A20 (Natoli *et al*, 2005; Hoffmann *et al*, 2006; Hayden and Ghosh, 2008). In this study, we examined E1A/Ras-transformed cells, in which A20 induction was abolished, and found that binding of Bcl-3, but not of other NF-*κ*B members, to the A20 promoter site was defective. As Bcl-3 protein expressions were abundant in both E1A/Ras and wild-type cells (Figure 7), the apparent, specific defect of Bcl-3 binding to the A20 gene regulatory site remains an interesting question for future studies. Interestingly, small Jab1 complex is reduced in Ras-transformed cells (Fukumoto *et al*, 2005). Deficiency of the Jab1 complex may contribute to the poor binding of Bcl-3 to the A20 promoter in E1A/Ras MEFs, as Jab1 was shown to enhance Bcl-3/p50 and the DNA complex (Dechend *et al*, 1999). Similar to Bcl-2, Bcl-3 was identified as an oncoprotein from chronic lymphocytic leukaemia with a t(14;19) chromosome translocation (McKeithan *et al*, 1990). As a member of the I*κ*B family of proteins, Bcl-3 was shown to be a transactivator for many genes, including cyclin D1, Bcl-2, and GATA-3 (Westerheide *et al*, 2001; Viatour *et al*, 2003; Corn *et al*, 2005; Massoumi *et al*, 2006). Similar to these Bcl-3 target genes, we showed here that Bcl-3 overexpression could induce A20 promoter activity and that both p52 and p50 collaborated with Bcl-3 for A20 induction (Figure 8). Identification of Bcl-3 and other potential regulators for A20 in transformed tumour cells may help advance our understanding of the control of cell life and death induced by death factors such as TNF.
